# Incidence trends and perinatal risk factors of developmental dysplasia of the hip: a nationwide population-based study from South Korea

**DOI:** 10.2340/17453674.2025.43980

**Published:** 2025-06-26

**Authors:** Kunhyung BAE, Jong Ho CHA, Jiyoung Agatha KIM, Soorack RYU, Jae Yoon NA, Young-Jin CHOI

**Affiliations:** 1 Department of Orthopaedic Surgery, Hanyang University Hospital, Hanyang University College of Medicine, Seoul; 2 Department of Pediatrics, Hanyang University Hospital, Seoul; 3 Department of Emergency Medicine, Seoul St. Mary’s Hospital, College of Medicine, The Catholic University of Korea, Seoul; 4 Biostatistical Consulting and Research Lab, Medical Research Collaborating Center, Hanyang University, Seoul; 5 Department of Pediatrics, College of Medicine, Hanyang University, Seoul; 6 Department of Pediatrics, Hanyang University Guri Hospital, Hanyang University College of Medicine, Guri, Republic of South Korea

## Abstract

**Background and purpose:**

South Korea has implemented the National Health Screening Program for Infants and Children (NHSPIC), which includes clinical hip screening with selective hip ultrasonography beginning at 4 months of age. We aimed to investigate the trends in developmental dysplasia of the hip (DDH), associated risk factors, and growth and motor developmental outcomes up to preschool age.

**Methods:**

We included a retrospective, population-based birth cohort of children born between 2008 and 2015. Patients diagnosed with DDH were stratified by age at detection (early diagnosed [<1 year] vs late diagnosed [≥1 year]) and treatment modalities (major surgery, minor surgery, or nonoperative). Growth and motor developmental outcomes were assessed using NHSPIC data collected up to 6 years of age.

**Results:**

Among 2,518,805 children, 4,854 (0.19%) were diagnosed with DDH. The incidence of DDH increased from 1.29 to 2.37 per 1,000 individuals, with the incidence of early diagnosed DDH increased from 0.70 to 1.94 per 1,000. However, the rate of surgical treatment remained unchanged (0.19–0.28 per 1,000). Children who underwent surgical treatment for DDH had a significantly higher incidences of short stature, and delayed gross motor development.

**Conclusion:**

After the introduction of the NHSPIC hip screening program, incidences of overall and early diagnosed DDH increased, whereas the surgical treatment rate showed no significant change. Surgical treatment for DDH was significantly associated with both short stature and delayed gross motor development.

Developmental dysplasia of the hip (DDH) is a major risk factor for hip osteoarthritis, manifesting as a broad spectrum of conditions affecting the hip joint [1]. Early diagnosis and timely treatment of DDH lead to a favorable prognosis, whereas delayed diagnosis is associated with a less favorable prognosis, patient morbidity, often requiring surgery, and higher medical expenses [2]. Therefore, early identification of DDH is important for restoring normal hip anatomy [3]. Despite efforts to establish hip screening for early diagnosis of DDH, controversy remains regarding the optimal method for early screening [4,5]. The choice between universal versus selective ultrasound screening is complex because of age-specific considerations for imaging and physical examination, variable risk factors, and concerns related to expenses [1]. Ultrasound screening is limited by interobserver variability due to its subjective interpretation [6]. The etiology of DDH is multifactorial, influenced by environmental and genetic predispositions [7]. While a Swedish study comprehensively evaluated prenatal, perinatal, and postnatal risk factors for DDH, nationwide studies encompassing various socioenvironmental and maternal factors influencing DDH are lacking [8]. Well-known risk factors for DDH include female sex, gestational diabetes mellitus (GDM), and breech presentation [9,10]. However, the underlying mechanisms linking these factors to DDH remain unclear and require further elucidation.

In South Korea, the National Health Screening Program for Infants and Children (NHSPIC) was initiated in 2007 for the entire pediatric population [11]. Universal clinical hip screening, followed by selective hip ultrasonography, was performed as part of this program and starts at 4 months of age. Analyzing this hip screening data may reveal its impact on the diagnosis and treatment of DDH. Additionally, as this dataset includes demographic and longitudinal growth information, it enables a comprehensive analysis of integrated associated risk factors and developmental outcomes.

We aimed to investigate the trends in incidence and treatment of DDH in early childhood using nationwide birth cohort data. In addition, we investigated the risk factors for DDH, and the preschool growth and motor development of patients stratified by their treatment methods.

## Methods

### Study design

This study investigated a retrospective birth cohort based on medical claims data from the National Health Insurance Service (NHIS) of the Republic of South Korea. This study conformed to the Strengthening the Reporting of Observational Studies in Epidemiology (STROBE) guidelines (Supplementary Table 1) [12].

### Database and national screening program for the pediatric population

The NHIS provides health insurance coverage to almost the entire South Korean population (97% of the population have health insurance and 3% are covered by medical aid). The NHIS collects the medical records of individuals, such as demographic information, medical visits, surgical records, and medical claims data.

In South Korea, the NHIS has implemented the National Health Screening Program for Infants and Children (NHSPIC), which consists of 7 rounds of annual health checkups by certified pediatricians during preschool ages (4–6, 9–12, 18–24, 30–36, 42–48, 54–60, and 66–71 months old). Assessments include body measurements, neurodevelopmental screening, and physical examinations, including hip screening. For this study, we created a birth cohort by linking results with the health claims data stored in the form of World Health Organization International Classification of Diseases, Tenth Revision (ICD-10) codes. Furthermore, we included medical claims data from individuals’ mothers and children’s NHSPIC results.

Hip screening was performed during the first round of NHSPIC using the Ortolani, Barlow, and Galeazzi tests, as well as an assessment of hip abduction limitation and asymmetrical hip fold. For reimbursement, physical examination findings for each organ system must be documented by each clinician. Children who require further diagnostic work-ups were referred to pediatric orthopedists.

### Study population

We investigated children born between 2008 and 2015 who had birth records and tracked their medical records until December 31, 2020 (n = 2,681,681). The exclusion criteria were as follows: (i) children diagnosed with cerebral palsy or profound motor impairment; (ii) children diagnosed with congenital malformation of the musculoskeletal system or chromosomal anomalies; (iii) children who underwent major hip fractures during infancy; and (iv) children who died during the observation period or had missing baseline information.

### Developmental dysplasia of the hip

The diagnosis of DDH is defined as follows: (i) individuals who had 3 or more orthopedic outpatient visits by 5 years of age with the affected diagnostic code or (ii) individuals who had more than 1 hospital visit with surgery for DDH by 5 years of age. The diagnostic code for DDH was ICD-10 code Q65, and the time of diagnosis was defined as the date of the first visit with the affected code [4]. Patients with DDH were divided into 2 groups: early diagnosed DDH, those who were diagnosed at < 1 year old and late diagnosed DDH, those who were diagnosed ≥ 1 year old [2].

Treatment of DDH was investigated using procedural codes and stratified into major surgery, minor surgery, and nonoperative groups. Major surgery was defined as open hip reduction, pelvic osteotomy, or femoral osteotomy. Minor surgery was defined as closed reduction of the hip and adductor tenotomy. Children who underwent both major and minor surgeries were classified into the major surgery group.

### Investigated variables

For demographic characteristics, sex, season at birth (spring: March through May, summer: June through August, autumn: September through November, and winter: December through February), socioeconomic status (SES: divided into quantiles base on the household expenditure on health insurance premium), and region of residence (metropolitan: ≥ 500,000 inhabitants, nonmetropolitan: < 500,000 inhabitants) were investigated. Maternal factors include GDM, pregnancy-induced hypertension (PIH), Caesarean section, breech presentation, oligohydramnios, dystocia, chorioamnionitis, and birth trauma. These conditions were defined when the mother was assigned the respective ICD-10 codes during pregnancy (Supplementary Table 2). For perinatal comorbidities, we included multiple conditions during pregnancy.

Finally, we linked the individual’s data from the first and second rounds of the NHSPIC (4–6 and 9–12 months of age) and selected multiple perinatal and postnatal variables (e.g., birth weight, primary milk feeding type, baby car seat, and baby walker) for the regression analysis. Annual growth measurements and developmental screening tests of the gross motor (GM) domain were included to track the longitudinal outcomes of children with DDH. Each body measurement (height, weight, and body mass index [BMI]) was converted into a Z-score [13]. The results of GM development scores are compared with those of the population of the same age, and screening was considered positive if the score fell below the cutoff of –1 standard deviation (SD), which warrants follow-up or diagnostic evaluation based on the Korean Developmental Screening Test for Infants & Children (K-DST) [14]. Detailed information regarding the variables used in this study is summarized in Supplementary Tables 2 and 3. The detailed gross motor developmental questionnaires included in the NHSPIC are described in Supplementary Table 4. The variables included in the study were predefined and stored within the NHIS database; therefore, additional validation could not be performed.

### Statistics

Comparisons of demographic characteristics between subgroups were analyzed using the chi-square test and Kruskal–Wallis test as appropriate. The cumulative incidence of DDH was calculated as a ratio of the number of newborns per year. Cumulative incidence was estimated with a 95% confidence interval (CI), assuming a Poisson distribution. We explored the risk factors for DDH using Cox proportional hazards regression analysis and expressed them as adjusted hazard ratios (aHRs) and 95% CIs. The proportional hazard assumption was confirmed through visual inspection of the log–log plot and assessment of non-significance with age. In adjusted model 1, we considered demographic characteristics (i.e., sex, SES, and season at birth). In model 2, we considered maternal and perinatal comorbidities. We included variables obtained from the NHSPIC, such as primary milk feeding type, baby car seat, baby walker, and birth weight (2.5 to 3.5 kg as reference) in model 3. Finally, significant variables in previous models were included in model 4. The growth and GM developmental status of children diagnosed with DDH were compared using the chi-square test and Kruskal–Wallis test as appropriate. Statistical significance was determined using 2-sided tests, with significance set at P value < 0.05. The statistical analyses were performed using SAS version 9.4 (SAS Institute Inc, Cary, NC, USA).

### Ethics, data sharing plan, funding, use of AI, and disclosures

The Institutional Review Board of our institution (IRB No. GURI 2024-04-003) exempted the study from having to obtain approval because we used only blinded public data to analyze and conduct the study. The data that supports the findings for this study is available to other researchers from the corresponding author upon reasonable request. This research was supported by a grant from Hanyang University (HY-202300000001168). No AI-assisted tools were used in the preparation or submission of this work. The authors received no financial or material support for the research, authorship, and/or publication of this article. Complete disclosure of interest forms according to ICMJE are available on the article page, doi: 10.2340/17453674.2025.43980

## Results

**Figure 1:**
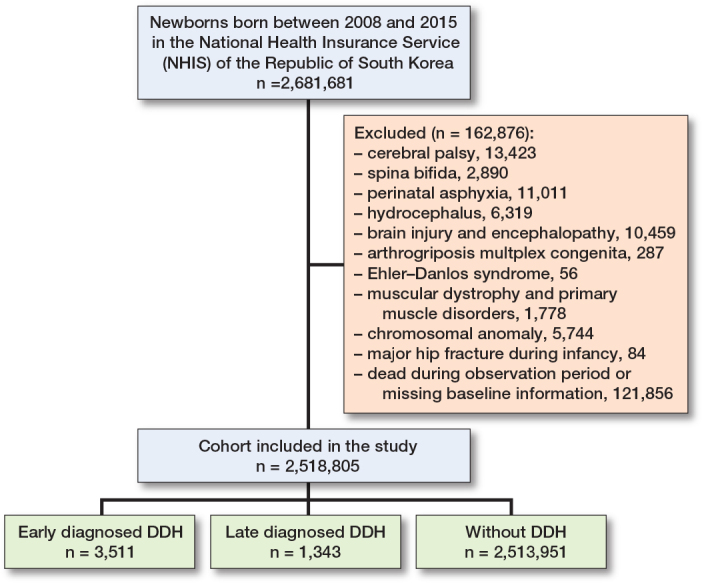
Flow diagram of study population selection process included in the birth cohort. DDH: developmental dysplasia of the hip.

2,518,805 children were included in the study, comprising 69% of the general pediatric population (Figure 1). 4,854 children were diagnosed with DDH (0.19%). Of these, 3,511 had early diagnosed DDH (0.14%) and 1,343 had late diagnosed DDH (0.05%). The median age of diagnosis was 0.4 years old (interquartile range [IQR] 0.2–0.5) for early diagnosed DDH, and 3.0 years old (IQR 1.5–5.3) for late diagnosed DDH. Children diagnosed with DDH revealed a significantly higher proportion of females, and higher SES (P < 0.001). All patients with DDH had a significantly higher incidence of maternal and perinatal comorbidities, such as GDM and breech presentation (P < 0.001). Patients with DDH had a higher participation rate in the initial NHSPIC rounds. The use of baby walkers during infancy was the highest in the group without DDH, followed by the late and early diagnosed DDH groups. Lastly, the proportion of exclusive breastfeeding was lowest in the early diagnosed DDH group (Table 1).

**Table 1:** Patient demographics, perinatal factors, maternal comorbidities, and treatment of DDH for all populations included from 2008 to 2015. Values are count (%) or median (Q1 to Q3) for continuous variables

Item	Early diagnosed DDH (n = 3,511)	Late diagnosed DDH (n = 1,343)	Without DDH (n = 2,513,951)	P value
*Demographic characteristics*				
Sex				< 0.001
Male	984 (28)	485 (36)	1,287,222 (51)	
Female	2,527 (72)	858 (64)	1,226,729 (49)	
SES				< 0.001
Q1	338 (9.6)	113 (8.4)	262,263 (10)	
Q2	625 (18)	240 (18)	542,029 (22)	
Q3	1,454 (41)	581 (43)	1,004,103 (40)	
Q4	1,094 (31)	409 (31)	705,556 (28)	
Residence**^a^**				< 0.001
Urban	2,615 (74)	959 (71)	1,709,467 (68)	
Rural	896 (26)	384 (29)	804,484 (32)	
Season at birth**^b^**				< 0.001
Spring	814 (23)	321 (24)	649,065 (26)	
Summer	796 (23)	315 (23)	614,328 (24)	
Autumn	949 (27)	362 (27)	630,240 (25)	
Winter	952 (27)	345 (26)	620,318 (25)	
*Perinatal factors and maternal comorbidities*				
Multiple birth	83 (2.4)	24 (1.8)	43,470 (1.7)	0.02
Preterm birth	309 (8.8)	85 (6.3)	111,518 (4.4)	< 0.001
IVF-ET	530 (15)	184 (14)	254,647 (10.)	< 0.001
IUGR	66 (1.9)	19 (1.4)	13,560 (0.5)	< 0.001
GDM	1,090 (31)	326 (24)	612,370 (24)	< 0.001
PIH	246 (7.0)	88 (6.6)	143,953 (5.7)	< 0.01
Cesarean section	1,093 (31)	388 (29)	570,584 (23)	< 0.001
Breech presentation	153 (4.4)	40 (3.0)	51,195 (2.0)	< 0.001
Oligohydramnios	38 (1.1)	12 (0.9)	12,352 (0.5)	< 0.001
Dystocia	282 (8.0)	110 (8.2)	176,898 (7.0)	0.01
Chorioamnionitis	199 (5.7)	78 (5.8)	107,954 (4.3)	< 0.001
Birth trauma	60 (1.7)	20 (1.5)	27,963 (1.1)	< 0.01
*NHSPIC results*				
Participation rates of				
1st round of NHSPIC				
(4–6 months old)	2,790 (80)	964 (72)	1,652,933 (69)	
2nd round of NHSPIC				
(9–12 months old)	2,351 (67)	780 (58)	1,285,906 (51)	
Birth weight, kg**^c^**	3.1	3.2	3.2	< 0.001
(Q1 to Q3)	(2.8 to 3.4)	(2.9 to 3.4)	(2.9 to 3.5)	
Primary milk feeding**^c^**				< 0.001
EBF	990 (36)	386 (40)	660,147 (40)	
EFF	1,225 (44)	425 (44)	664,137 (40)	
PBF	573 (21)	153 (16)	326,156 (20)	
Baby car seat**^c^**	2,004 (72)	716 (74)	1,162,063 (70)	< 0.01
Baby walker**^d^**	1,344 (48)	539 (56)	938,778 (57)	< 0.001
Body measurement**^c^**				
Height-at-age Z-score	0.6	0.6	0.7	< 0.001
(Q1 to Q3)	(–0.1 to 1.2)	(–0.1 to 1.3)	(–0.0 to 1.3)	
Weight-at-age Z-score	0.7	0.7	0.7	< 0.001
(Q1 to Q3)	(0.0 to 1.2)	(0.1 to 1.3)	(0.1 to 1.3)	
Gross motor development				
< –1 SD**^d,e,f^**	219 (9.8)	65 (10)	61,354 (5.4)	< 0.001
*Treatment of DDH*				
Major surgery	58 (1.7)	254 (19)	n/a	<0.001
Minor surgery	134 (3.8)	134 (10)	n/a	<0.001
Nonoperative	3,319 (95)	955 (71)	n/a	<0.001

DDH: developmental dysplasia of hip; SES: socioeconomic status; Q: quartile; IVF-ET: in vitro fertilization embryo transfer; IUGR: intrauterine growth retardation; GDM: gestational diabetes mellitus; PIH: pregnancy induced hypertension; n/a: not applicable: NHSPIC: National Health Screening Program for Infants and Children; EBF: exclusive breastfeeding; EFF: exclusive formula feeding; PBF: partial breastfeeding; SD: standard deviation.

a Urban: ≥ 500,000 inhabitants, Rural: < 500,000 inhabitants.

b Spring: March, April, May; Summer: June, July, August; Autumn: September, October, November; Winter: December, January, February.

c Obtained from the 1st round of NHSPIC (4–6 months old).

d Obtained from the 2nd round of NHSPIC (9–12 months old).

e Obtained from either from either Korean Developmental Screening Test (K-DST) or Korean Ages & Stage Questionnaires (K-ASQ).

f For those who had gross motor domain < –1 SD, close observation or diagnostic evaluation is recommended.

### Incidence and treatment trend of DDH

**Figure 2:**
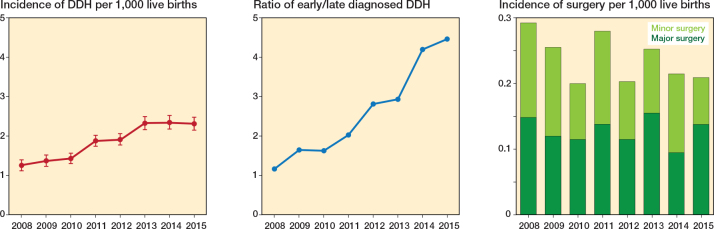
Line charts indicate the 8-year trend of the overall incidence of DDH (red line), and ratio of early diagnosed DDH/late diagnosed DDH (blue line). Red filled circles indicate annual incidence and red vertical lines indicate 95% confidence interval. Stacked bar chart presents the 8-year trend of the incidence of major surgery and minor surgery stratified according to birth year. DDH: developmental dysplasia of the hip

The incidence of DDH gradually increased, from 1.29 in 2008 to 2.37 in 2015 (per 1,000 persons, P for trend < 0.001) (Figure 2). The ratio of early diagnosed/late diagnosed DDH also increased from 1.19 in 2008 to 4.57 in 2015. During the study period, 312 patients underwent major surgery and 268 underwent minor surgery. Patients with late diagnosed DDH had a significantly higher incidence of surgeries (major: 254 cases, 18.9%; minor: 134 cases, 10.0%) than those with early diagnosed DDH (major: 58 cases, 1.7%; minor: 134 cases, 3.8%) (P < 0.001) (Table 1). Conversely, the temporal tread of the overall incidences of major and minor surgeries plateaued, reaching 0.19 to 0.28 cases per 1,000 persons, in contrast to an increasing number of diagnoses (Figure 2).

**Figure 3:**
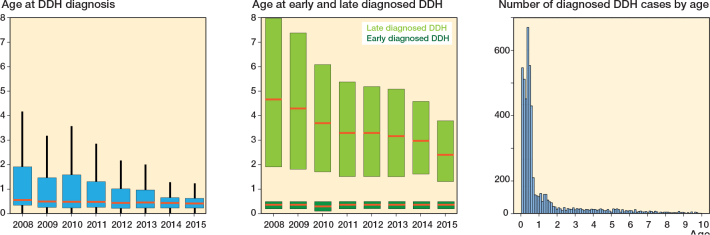
Box plot presenting the age at DDH diagnosis (blue box), late diagnosed DDH (light green box), and early diagnosed DDH (dark green box). Bar graph presenting the number of diagnosed DDH cases by birth year. The red horizontal line represents the median value, while the top and bottom of each box indicate Q3 and Q1, respectively. DDH: developmental dysplasia of the hip.

During the observation period, the median age of early diagnosed DDH was stationary at 0.3 years old, whereas the median age of late diagnosed DDH cases gradually decreased from 4.1 (IQR 1.9–8.0) to 2.7 (IQR 1.6–4.6) years old (Figure 3).

### Regression models for predicting risks of DDH

The remaining significant risk factors for DDH were female sex among the demographic characteristics (model 1), GDM and breech presentation among the maternal and perinatal variables (model 2), and primary milk feeding type and use of baby walkers during infancy as postnatal variables (model 3) (Table 2).

**Table 2:** Regression analysis predicting the risk factors of DDH according to demographics, maternal comorbidities, perinatal factors, and results of 1st and 2nd round of NHSPIC. Numbers are expressed as HR and 95% confidence interval (CI)

Item	Model 1 HR (CI)	Model 2 HR (CI)	Model 3 HR (CI)	Model 4 HR (CI)
Sex (male as ref.)	1.26 (1.19–1.35) **^a^**			1.27 (1.18–1.36) **^a^**
SES (Q1 as ref.)				
Q2	0.95 (0.85–1.07)			
Q3	0.93 (0.84–1.03)			
Q4	0.97 (0.87–1.08)			
Urban (rural as ref.)	1.01 (0.95–1.08)			
Season at birth (spring as ref.)				
Summer	1.03 (0.95–1.12)			
Autumn	1.04 (0.96–1.13)			
Winter	1.05 (0.97–1.14)			
Multiple birth		1.00 (0.82–1.23)		
Preterm birth		1.08 (0.96–1.20)		
IVF-ET		1.01 (0.93–1.10)		
IUGR		1.08 (0.86–1.35)		
GDM		1.19 (1.11–1.27) **^a^**		1.17 (1.10–1.26) **^a^**
PIH		1.00 (0.89–1.12)		
Cesarean section		1.04 (0.98–1.11)		
Breech presentation		1.43 (1.23–1.65) **^a^**		1.40 (1.19–1.65) **^a^**
Oligohydramnios		1.01 (0.75–1.36)		
Dystocia		0.95 (0.85–1.06)		
Chorioamnionitis		0.93 (0.82–1.06)		
Birth trauma		1.18 (0.94–1.47)		
Primary milk feeding (EBF as ref.)				
EFF			1.12 (1.04–1.20) **^a^**	1.10 (1.02–1.18) **^a^**
PBF			1.19 (1.08–1.30) **^a^**	1.19 (1.09–1.20) **^a^**
Baby car seat**^b^**			0.93 (0.87–1.004)	
Baby walker**^b^**			0.86 (0.81–0.92) **^a^**	0.88 (0.84–0.94) **^a^**
Birth weight (2.5–3.5 kg as ref.)				
< 2.5 kg			1.00 (0.88–1.14)	
3.5–4.5 kg			0.99 (0.91–1.07)	
≥ 4.5 kg			1.24 (0.62–2.48)	

For abbreviations, see Table 1, and ref: reference.

a Statistically significant

b Obtained from the NHSPIC

In model 4, which considered variables that remained significant, breech presentation (aHR 1.40, CI 1.19–1.65) was the most significant risk factor, followed by female sex (aHR 1.27, CI 1.18–1.36), and GDM (aHR 1.17, CI 1.10–1.26). Compared with children with exclusive breastfeeding, formula-fed children had an elevated risk of DDH (partial breastfeeding, aHR 1.19, CI 1.09–1.20; exclusive formula feeding, aHR 1.10, CI 1.02–1.18). Children who used a baby walker during infancy had a lower risk of developing DDH than nonusers (aHR 0.88, CI 0.84–0.94).

### Growth and GM development outcomes

Children who underwent major surgery had the highest incidence of short stature and were underweight, followed by the minor surgery group and the nonoperative group (7.9% vs 3.9% vs 3.1% for short stature by 48 months of age, P = 0.02) (Supplementary Table 5). In terms of GM development delay, the incidence of screening positivity was highest in the major surgery group (16.0% in 18–24 months, 9.4% in 30–48 months), followed by the minor surgery group (P < 0.001).

## Discussion

This is the first study to analyze nationwide population-based data from more than 2 million births to determine trends in the incidence, diagnosis, and treatment of DDH, including comprehensive perinatal risk factors and long-term developmental outcomes in patients who underwent surgery.

We investigated trends in the overall incidence and treatment of DDH in children born between 2008 and 2015 who underwent universal clinical screening and selective ultrasonography since 2007. Furthermore, we analyzed the risk factors for DDH and growth outcomes of patients with DDH. The incidence of DDH increased from 1.29/1,000 in 2008 to 2.37/1,000 in 2015, with a corresponding increase in the early diagnosed DDH/late diagnosed DDH ratio. However, the overall incidence of major and minor surgeries remained 0.19 to 0.28 per 1,000 live births.

### Incidence and treatment trend of DDH

Our study found that the overall incidence of DDH was 1.93 per 1,000 live births. Previous studies have reported varying nationwide incidence rates, ranging from 0.12 in Sweden, 1.54 in Taiwan, and 7.6 in Japan, to 26 in Austria per 1,000 live births [4,8,15,16]. We assume that discrepancies in incidence rates result from methodological differences. The Swedish study analyzed data from a prospective registry maintained by pediatric orthopedic surgeons, whereas the studies from Taiwan, Japan, and Austria identified DDH cases using designated ICD-10 codes. As we could not assess the clinical manifestations of the affected cases, the diagnostic reliability could not be evaluated with our study design.

The optimal strategy for DDH screening has not yet been validated, with considerable variations across countries. Various screening methods, including universal ultrasonographic screening [17], selective ultrasonographic screening [18,19], and clinical screening [4], are applied. However, a recent meta-analysis found no significant differences in late detection and surgical treatment rates between these methods, suggesting that universal screening may contribute to overdiagnosis and reduced cost-effectiveness. Following the implementation of universal clinical screening and selective ultrasonography, the incidence of both overall and early diagnosed DDH has increased in South Korea (see Figure 2). This could be explained by the implementation of nationwide screening programs following the participation of pediatricians, which may have heightened the awareness of DDH and subsequently increased diagnosis rates [20,21]. Additionally, certain clinical screening criteria—particularly asymmetric thigh folds—may also play a role. Although asymmetric thigh folds have traditionally been considered a screening factor for DDH in some Asian countries [22,23], Kang et al. reported that this finding may not have significant clinical relevance in DDH screening [24]. Despite the increasing incidence of DDH, the surgical rate remained relatively stable, raising concerns regarding the effectiveness of screening strategies. One possible explanation is the relatively late initiation of the screening program at 4 months of age, which may reduce the effectiveness of early treatment. In South Korea, hip screening begins later than in many Western countries, where earlier screening is associated with lower surgical rates [16,25].

### Regression models for predicting risks of DDH

Our risk factor analysis investigated various maternal, perinatal, and postnatal factors that may be significantly associated with DDH. Interestingly, among postnatal variables, we showed that the use of a baby walker and exclusive breastfeeding were protective against DDH. Swaddling or postures with extension and adductions during infancy promote DDH. This may be explained by a baby walker and the positioning associated with breastfeeding may promote hip joint abduction and flexion when the infant is seated.

### Growth and GM development outcomes

Our study found that surgically treated patients, especially those who underwent major surgery for DDH, had a higher incidence of short stature, underweight, and GM delay than those who did not. Considering that previous prognostic studies primarily focused on hip-related outcomes, our findings emphasize the importance of monitoring postoperative growth to support catch-up growth. One possible explanation is that prolonged hip immobilization following DDH surgery can lead to delayed rehabilitation, muscle atrophy, and impaired motor function. Although the GM development assessment tools used in this study were intended for screening, our results emphasize the need for regular surveillance of GM development and long-term postoperative care and comprehensive postoperative care. Importantly, previous research has also shown an association between major surgeries with developmental delay [26,27]. Therefore, reinforcement of the necessity of a multidisciplinary approach involving pediatric orthopedic surgeons, general pediatricians, and rehabilitation specialists is required. Targeted interventions, such as nutritional support, rehabilitation programs, and structured exercise regimens, may be beneficial for optimizing recovery. However, our findings should not be misinterpreted as evidence that DDH surgery leads to permanent growth stunting or GM delay. Further longitudinal studies are required to determine the long-term effects of surgical treatment on overall growth and motor function.

### Limitations

First, definition of DDH was based on diagnostic codes and hospital visits. Although diagnostic codes were entered by certified pediatricians and orthopedic surgeons, detailed sonographic findings data was not validated. To qualify our diagnoses, we limited the confirmed diagnoses of DDH to patients who had repeated clinic visits or had undergone surgical procedures. However, compared with direct review of medical records and imaging studies, this approach may contain inaccuracies in diagnosis and its timing. Second, this study did not provide detailed information for the nonoperative group. In South Korea, nonoperative treatment modalities such as Pavlik’s harness and hip abduction braces are not covered by health insurance. Third, owing to the innate limitations of our data, we could not investigate known risk factors for DDH, including family history, order of birth, and hip laterality.

### Conclusion

Since the implementation of universal hip screening in South Korea, there has been an increase in early treatment; however, surgical treatment rates have remained unchanged. Surgical treatment for DDH was significantly associated with both short stature and delayed gross motor development. Furthermore, children who underwent major surgery showed GM development delays, emphasizing the need for long-term postoperative monitoring and multidisciplinary care.

*In perspective,* establishment of optimal screening strategies is essential to improve early detection and long-term developmental outcome.

### Supplementary data

Supplementary Tables 1–5 are available as Supplementary data on the article page, doi: 10.2340/17453674.2025.43980

## Supplementary Material



## Notes

BK and JHC developed the study concept and design. JYN and Y-JC designed and supervised the study. JHC and SR collected data and conducted the study and performed the statistical analyses. BK, JHC, and JAK wrote the draft of the manuscript. JYN and Y-JC critically revised the manuscript.

Handling co-editors: Ilkka Helenius and Robin Christensen

*Acta* thanks Peter John Cundy, Yrjänä Nietosvaara, and Carl Johan Tiderius for help with peer review of this manuscript.
