# Supplementary material for: Incidence trends and perinatal risk factors of developmental dysplasia of the hip: a nationwide population-based study from South Korea
Source: Acta Orthop. 2025 Jun 26;96:477–84. doi: 10.2340/17453674.2025.43980 (PMC12198684; doi:10.2340/17453674.2025.43980)
Supplement: Supplementary file 1 [file ActaO-96-43980-s1.pdf]

Supplementary data

Supplementary Table 1. STROBE statement—Checklist of items that should be included in reports of cohort studies

	Item No	Recommendation	Page No
Title and abstract	1	(*a*) Indicate the study's design with a commonly used term in the title or the abstract	p1-3
		(*b*) Provide in the abstract an informative and balanced summary of what was done and what was found	p1-3
Introduction			
Background/rationale	2	Explain the scientific background and rationale for the investigation being reported	Introduction, p4
Objectives	3	State specific objectives, including any prespecified hypotheses	Introduction, p4
Methods			
Study design	4	Present key elements of study design early in the paper	Methods, p5-8
Setting	5	Describe the setting, locations, and relevant dates, including periods of recruitment, exposure, follow-up, and data collection	Methods, p5-8
Participants	6	(*a*) Give the eligibility criteria, and the sources and methods of selection of participants. Describe methods of follow-up	Methods, p5-8
		(*b*) For matched studies, give matching criteria and number of exposed and unexposed	Methods, p5-8
Variables	7	Clearly define all outcomes, exposures, predictors, potential confounders, and effect modifiers. Give diagnostic criteria, if applicable	Methods, p5-8
Data sources/ measurement	8*	For each variable of interest, give sources of data and details of methods of assessment (measurement). Describe comparability of assessment methods if there is more than one group	Methods, p5-8
Bias	9	Describe any efforts to address potential sources of bias	Methods, p5-8
Study size	10	Explain how the study size was arrived at	Methods, p5-8
Quantitative variables	11	Explain how quantitative variables were handled in the analyses. If applicable, describe which groupings were chosen and why	Methods, p5-8
Statistical methods	12	(*a*) Describe all statistical methods, including those used to control for confounding	Methods, p5-8
		(*b*) Describe any methods used to examine subgroups and interactions	Methods, p5-8
		(*c*) Explain how missing data were addressed	Methods, p5-8
		(*d*) If applicable, explain how loss to follow-up was addressed	Methods, p5-8
		(*e*) Describe any sensitivity analyses	Methods, p5-8
Results			
Participants	13*	(a) Report numbers of individuals at each stage of study—eg numbers potentially eligible, examined for eligibility, confirmed eligible, included in the study, completing follow-up, and analysed	Results, p8-10, Figure 1

		(b) Give reasons for non-participation at each stage<br>(c) Consider use of a flow diagram	
Descriptive data	14*	(a) Give characteristics of study participants (eg demographic, clinical, social) and information on exposures and potential confounders<br>(b) Indicate number of participants with missing data for each variable of interest<br>(c) Summarise follow-up time (eg, average and total amount)	Results, p8-10<br><br>Results, p8-10
Outcome data	15*	Report numbers of outcome events or summary measures over time	Results, p8-10
Main results	16	(*a*) Give unadjusted estimates and, if applicable, confounder-adjusted estimates and their precision (eg, 95% confidence interval). Make clear which confounders were adjusted for and why they were included<br>(*b*) Report category boundaries when continuous variables were categorized<br>(*c*) If relevant, consider translating estimates of relative risk into absolute risk for a meaningful time period	Results, p8-10<br><br>Results, p8-10<br>Results, p8-10
Other analyses	17	Report other analyses done—eg analyses of subgroups and interactions, and sensitivity analyses	Results, p8-10
Discussion			
Key results	18	Summarize key results with reference to study objectives	Discussion, p10-14
Limitations	19	Discuss limitations of the study, taking into account sources of potential bias or imprecision. Discuss both direction and magnitude of any potential bias	Discussion, p10-14
Interpretation	20	Give a cautious overall interpretation of results considering objectives, limitations, multiplicity of analyses, results from similar studies, and other relevant evidence	Discussion, p10-14
Generalizability	21	Discuss the generalizability (external validity) of the study results	Discussion, p10-14
Other information			
Funding	22	Indicate the source of funding and the role of the funders in the present study and, if applicable, for the original study on which the present article is based	p15

*Provide information separately for exposed and unexposed groups.

Note: An Explanation and Elaboration article discusses each checklist item and gives methodological background and published examples of transparent reporting. The STROBE checklist is best used in conjunction with this article (freely available on the Web sites of PLoS Medicine at http://www.plosmedicine.org/, Annals of Internal Medicine at http://www.annals.org/, and Epidemiology at http://www.epidem.com/). Information on the STROBE Initiative is available at http://www.strobe-statement.org.

Supplementary Table 2. Detailed information of variables included in the study

Variables	Definition
**A. Maternal and Perinatal variables included in the study**	
Multiple birth	Z372,Z373,Z375,Z376,Z383,Z384,Z385,Z386,Z387,Z388[a]
Preterm birth	P07[a]
IVF-ET	N97[b]
IUGR	P05[b]
GDM	O244 to O249[b]
PIH	O141 to O149[b]
Cesarean section	O82[b]
Breech presentation	1. O321, O641, O801, O830, O831[b]<br>2. P017, P030[a]
Oligohydroamnios	1. O40, O410[b]<br>2. P012[a]
Dystocia	O64 to O66[b]
Chorioamnionitis	O41[b]
Birth trauma	P10 to P15[b]
**B. Variables obtained from NHSPIC**	
Primary milk feeding type	What is your baby's primary milk feeding type?<br>a) exclusive breastmilk, b) exclusive formula milk, c) mixed feeding (breastfeeding and formula), d) specialized milk
Baby car seat	Q. Where do you install your baby's car seat?<br>a) at the back seat, b) at the front seat, c) I don't use baby car seat
Baby walker	Q. Do you use baby walker?<br>a) yes, b) no
Birth weight	Q. What was your child's birth weight? Please describe the birth weight to the nearest 0.1 kg
Body measurements - height	a) Height of children below 24 months who could not stand on their own was measured using an infantometer. Measured to the nearest 0.1cm<br>b) Height of children from 24 months of age with the child wearing a comfortable clothing without shoes and standing upright and the child's hips perpendicular to the central axis, heels on a footrest, and head on a Frankfurt plane. Measured to the nearest 0.1cm
Body measurements - weight	a) Weight of children below 24 months who could not stand on their own was measured using an infantometer. Measured to the nearest 0.1kg<br>b) Weight of children from 24 months was measured using an electric scale with the child wearing a comfortable clothing. Measured to the nearest 0.1kg
Body measurements - BMI	BMI was calculated as weight divided by height in meters squared.
Gross motor development	Parents fill out a questionnaire based on their child's months of age, with eight questions for each domain. Each answer is given on 4-point Likert scale indicating "Doing very well', "Can do", "Not doing well", and "Cannot do". The summed scores for each domain are categorized into; (1) normal development (summed score ≥ –1 SD), and (2) screen positive (summed score < –1 SD)

Abbreviations: ICD-10, International Classification of Diseases, Tenth Revision; NHSPIC, the National Health Screening Program for Infants and Children; IVF-EF, in vitro fertilization-embryo transfer; IUGR, intrauterine growth retardation; GDM, gestational diabetes mellitus; PIH, pregnancy-induced hypertension.. [a] Defined as when a child is assigned with respective ICD-10 codes within first year of life. [b] Defined as when child's mother is assigned with respective ICD-10 codes during pregnancy.

Supplementary Table 3. Patient criteria, detailed information of ICD-10 and procedural codes for study population

Variables	Definition [a]
**Exclusion criteria 1**. Children diagnosed with cerebral palsy or certain conditions that could result in motor impairment	
Cerebral palsy	G80 to G82
Spina bifida	Q05
Perinatal asphyxia	P20 to P21
Hydrocephalus	Q03, G91, P917
Brain injury and encephalopathy [b]	G93
**Exclusion criteria 2**. Children diagnosed with congenital malformation of the musculoskeletal system or chromosomal anomalies	
Arthrogryposis multiplex congenita	Q74.3
Ehler Danlos syndrome	Q79.6
Muscular dystrophy and primary muscle disorders	G71 to G72
Chromosomal anomaly	Q9
**Exclusion criteria 3**. Children underwent major hip fractures during infancy	
Femur neck fracture	S72.0
Pertrochanteric fracture	S72.1
Acetabular fracture	S32.4
**Exclusion criteria 4.** Children expired during observation period or missing baseline information	
**Diagnoses of DDH**	Q65
**Major surgery for DDH**	
Osteotomy of extremities	N0302
Osteotomy of pelvis	N0303
Femur osteotomy and internal fixation	N0305
Open reduction of dislocation of hip	N0751
**Minor surgery for DDH**	
Closed reduction of congenital hip dislocation	N0811, N0812
Closed reduction of hip dislocation	N0761
Adductor tenotomy	N0931, N0911, N0912

Abbreviations: ICD-10, International Classification of Diseases, Tenth Revision; DDH, developmental dysplasia of hip.

[a] Exclusion criteria is based on ICD-10 codes assigned in the National Health Insurance Service database

[b] Surgical records are assigned with codes registered in the National Health Insurance Service database

Supplementary Table 4. Gross motor developmental questionnaires included in the NHSPIC.

**Baseline demographic questions**	1. Baby's name<br>2. Baby's date of birth (If preterm birth, expected date of confinement)<br>3. Baby's sex<br>4. Person filling out the questionnaire<br>5. Mother's age and degree of education<br>6. Father's age and degree of education
**Developmental questionnaires [a]:**<br>• The questions below are to assess your child's ability to perform different actions. If your child can perform a behavior, but hasn't been doing it often, it should be considered as 'available'.<br>• Try each activity with your baby before answering.<br>• For each question, please indicate one of the four answers; "Doing very well', "Can do", "Not doing well", "Cannot do".	
**Gross motor skills at 4–5 months old**	1. While your baby is on his back, can your baby turn over halfway?<br>2. When your baby is on his/her tummy, can your baby raise his head up briefly?<br>3. When you pull him/her up by the arms when your baby is lying down, does his/her neck follow you, instead of falling behind?<br>4. When your baby is on his//her tummy, does your baby lift his/her chest and support himself/herself with arms?<br>5. When your baby is on his/her tummy, does your baby flip to supine?<br>6. While your baby is on his back, does your baby flip from supine to prone?<br>7. Does your baby grab his/her own feet and plays alone when lying down?<br>8. When you hold him in a sitting position, does your baby sits unassisted for at least 30 seconds with both hands on the floor?
**Gross motor skills at 12–13 months old**	1. While holding onto furniture, does your baby lower him/herself with control (without falling or flopping down)?<br>2. Does your baby walk beside furniture while holding onto it with both hands?<br>3. Does your baby stand without any help for more than 5 seconds?<br>4. Does your baby walk beside furniture while holding on with only one hand?<br>5. Does your baby stand up on his/her own without holding onto anything?<br>6. If you hold both hands just to balance your baby, does he/she take few steps without falling? (if your baby already walks alone, please mark "yes".)<br>7. Does your baby take two or three steps alone?<br>8. Does your baby take about ten steps alone?
**Gross motor skills at 22–23 months old**	1. Does your child step backwards?<br>2. Does your child kick a large ball standing still by swing his leg forward?<br>3. Does your child walk down stairs one by one while holding onto the rail?<br>4. Does your child jump with both feet off the ground?<br>5. Does your child jump to the ground with both feet together at the stairs?<br>6. Does your child throw a ball overhead while standing still?<br>7. Does your child walk up the stairs with holding onto the rail?<br>8. Does your child walk more than four steps on tiptoes?
**Gross motor skills at 33–35 months old**	1. Does your child stand on one foot for about 1 second without holding onto anything?<br>2. Does your child walk down stairs, placing both feet together on one step without holding onto anything?<br>3. Does your child walk up stairs, using only one foot on each stair?<br>4. Does your child catch a big ball with both arms and chest?<br>5. Does your child pedal a tricycle to move forward?<br>6. Does your child walk straight ahead along a line?<br>7. Does your child perform a long jump with both feet together in place?<br>8. Does your child stands alone more than 3 seconds on one feet without holding onto anything?
**Gross motor skills at 48–53 months old**	1. Does your child stands alone more than 3 seconds on one feet without holding onto anything?<br>2. Does your child ride a tricycle with training wheels?

	3. Does your child hop two or three steps on one feet?<br>4. While standing, does your child throw a ball overhead by raising his arm to shoulder height and throwing the ball forward?<br>5. Without holding onto anything, does your child walk down stairs with one step at a time?<br>6. Does your child stop a rolling ball with his/her own feet?<br>7. Does your child catch a tennis ball flying from a distance of 2 meters with both hands?<br>8. Does your child bounce the ball on the floor?

Abbreviations: K-DST; Korean Developmental Screening Test for Infants & Children; GM, gross motor.

[a] The K-DST consists of developmental questionnaires across six developmental domains: gross motor, fine motor, cognition, language, social skills, and self-help. Parents fill out a questionnaire based on their child's months of age, with eight questions for each domain. Each answer is given on 4-point Likert scale indicating "Doing very well', "Can do", "Not doing well", and "Cannot do", respectively. The summed scores for each domain are classified with certain levels; categorized into; (1) normal development (summed score ≥ -1 SD [standard deviation]), (2) recommendation to follow-up (borderline, defined as summed score < -1 SD and ≥ -2 SD), and recommendation to professional evaluation (screen-positive, defined as summed score < -2 SD).

Supplementary Table 5. Growth and gross motor developmental status of patients with DDH according to treatment methods in 3rd (18 to 24 months), 4th (30 to 36 months) and 5th (42 to 48 months) round of NHSPIC

	**Major surgery (n = 312)**	**Minor surgery (n = 268)**	**Non-operative (n = 4274)**	***p* value**
**Participation rates of 3rd round of NHSPIC (18 to 24 months)**	199 (63.8%)	177 (66.0%)	3509 (82.1%)	
**Body measurements**				
**Short stature (HAZ < -2SD)**	6 (3.0%)	3 (1.7%)	47 (1.3%)	0.15
**Underweight (WAZ < -2SD)**	4 (2.0%)	1 (0.6%)	14 (0.4%)	< 0.01
**Gross motor development [a]**				
**Recommend for follow-up (< -1 SD)**	26 (16.0%)	20 (14.7%)	106 (3.5%)	< 0.001
**Participation rates of 4th round of NHSPIC (30 to 36 months)**	218 (69.9%)	204 (76.1%)	3450 (80.7%)	
**Participation rate of 5th round of NHSPIC (42 to 48 months)**	193 (61.9%)	183 (68.3%)	2784 (65.1%)	
**Body measurements [b]**				
**Short stature (HAZ < -2SD)**	13 (4.9%)	11 (4.7%)	99 (2.6%)	0.02
**Underweight (WAZ < -2SD)**	21 (7.9%)	9 (3.9%)	119 (3.1%)	< 0.001
**Failure to thrive (BMIAZ < -2SD)**	8 (3.0%)	7 (3.0%)	88 (2.3%)	0.62
**Gross motor development [a,b]**				
**Recommend for follow-up (< -1 SD) [c]**	24 (9.4%)	11 (5.0%)	115 (3.1%)	< 0.001

Numbers are expressed as n (%).
Abbreviations: DDH, developmental dysplasia of hip; NHSPIC; National Health Screening Program for Infants and Children; HAZ, height-at-age Z-score; WAZ, weight-at-age Z-score; BMIAZ, body mass index–at-age Z-score; SD, standard deviation.
[a] Obtained from either Developmental screening tests from either Korean Developmental Screening Test (K-DST) or Korean Ages & Stage Questionnaires (K-ASQ).
[b] If data from the 5th round NHSPIC is missing, data from the 4th round NHSPIC has been selected.
[c] For those who had gross motor domain < -1SD, close observation or diagnostic evaluation is recommended.
